# The Dark Side of Employee’s Leadership Potential: Its Impact on Leader Jealousy and Ostracism

**DOI:** 10.3390/bs15081001

**Published:** 2025-07-23

**Authors:** Zhen Yu, Feiwen Wang, Long Ye, Ganli Liao, Qichao Zhang

**Affiliations:** 1 School of Economics and Management, Beijing Jiaotong University, Beijing 100044, China; 19113077@bjtu.edu.cn (Z.Y.); yelong@bjtu.edu.cn (L.Y.); 2 Business School, Beijing Information Science and Technology University, Beijing 102206, China; 2023020786@bistu.edu.cn (F.W.); glliao@bistu.edu (G.L.)

**Keywords:** employee’s leadership potential, leader jealousy, leader ostracism, organizational competitive climate, leader’s core self-evaluation

## Abstract

In today’s rapidly evolving organizations, talent management plays a critical role in driving sustainable growth. Talents, particularly those exhibiting leadership potential, are often seen as essential assets for organizational development. However, the presence of high employee’s leadership potential can also generate adverse emotional reactions from leaders, potentially leading to behaviors such as leader jealousy and leader ostracism. This study investigates the dark side of employee’s leadership potential by examining the mechanisms through which employee’s leadership potential influences leader ostracism, with leader jealousy acting as a mediator. Drawing on social comparison theory, we propose a theoretical model that includes organizational competitive climate and leader’s core self-evaluation as moderating factors. Using a three-wave survey of 672 leaders in the Chinese construction industry, hierarchical regression analysis was employed to test the hypotheses. The results show that employee’s leadership potential significantly increases both leader jealousy and leader ostracism, with leader jealousy serving as a mediator. Moreover, a high organizational competitive climate strengthens the relationship between employee’s leadership potential and leader jealousy, thereby enhancing the entire mediated effect. In contrast, high leader core self-evaluation weakens the relationship between employee’s leadership potential and leader jealousy, reducing the likelihood of leader ostracism and attenuating the mediated effect. This study provides both theoretical contributions and practical insights for organizations seeking to manage high-leadership potential employees while minimizing the risk of negative leadership behaviors.

## 1. Introduction

Talent has long been considered a fundamental driver of sustained organizational competitiveness (Harsch & Festing, 2020; Kossyva et al., 2024). As digital transformation and the knowledge-based economy accelerate, the demand for high-potential talent is growing, with employees exhibiting high-leadership potential increasingly recognized as critical resources for organizational development and change (Groves & Feyerherm, 2022; Iles, 1997). Employee’s leadership potential (ELP) refers to an employee’s potential to progress from a rank-and-file position to a mid- or senior-level manager, encompassing their ability to lead, manage, and influence others (Dries & Pepermans, 2012; Troth & Gyetvey, 2014). The literature consistently highlights the critical role of high-potential employees in driving organizational innovation, strategic change, and sustained growth (Groves & Feyerherm, 2022; Iles, 1997). These employees are often seen as key drivers of future organizational success and receive increased resources and attention (Dries & Pepermans, 2012). However, is ELP solely positive? In reality, it can also pose a potential threat to the status and resources of current leaders (Kotlyar et al., 2014) and become a source of risk by triggering negative leader emotions, damaging supervisor–subordinate relationships, and contributing to talent attrition (Xue et al., 2020). This paper, therefore, explores the dark side of ELP, reveals its potential negative impacts, and provides both theoretical insights and practical guidance for effectively managing ELP.

Existing research has rarely examined the psychological and behavioral responses of ELP from the perspective of their direct supervisors, with most studies focusing on ELP identification criteria or analyzing its role from the perspectives of employees and coworkers (Dries & Pepermans, 2012; Troth & Gyetvey, 2014). Leader ostracism refers to a leader’s intentional or unintentional, overt or covert interpersonal deviant behaviors, such as ignoring, rejecting, and ostracizing subordinates (Howard et al., 2020; Ren et al., 2018). Recent studies have found that leader ostracism is not limited to underperforming or disciplined employees but may also target high-potential employees who are performing well, such as stars, high-performing and creative individuals (Carnevale et al., 2024; Howard et al., 2020; H. Liu et al., 2021). As a specific form of workplace cold violence within organizations, leader ostracism has become a common method for maintaining personal authority and mitigating interpersonal conflicts due to its mild and conservative nature (Azeem et al., 2024). However, although leader ostracism appears nonviolent, this behavior can negatively impact the developmental motivation, team cohesion, and innovative vitality of high-leadership potential employees (Henle et al., 2023; Lam et al., 2011). Therefore, this paper will further explore how ELP triggers leader ostracism by influencing leaders’ psychological responses. Understanding the mechanisms between the two will provide crucial insights into the relationship between leaders and high-potential employees, which is vital for both theoretical advancement and managerial practice.

This study introduces social comparison theory (SCT), which posits that the impact of ELP on leader ostracism primarily operates through the central mechanism of leader jealousy. Leader jealousy refers to the negative emotions leaders experience when they perceive that others possess important resources (e.g., competence, perspectives, or power) that they desire but lack, within the context of social comparison (Bani-Melhem et al., 2023). Leader jealousy is often accompanied by feelings of threat, inferiority, and concern over the loss of status or relationships (C. Liu et al., 2024). A large body of literature links jealousy to injurious behaviors in employees (Duffy et al., 2012; E. Kim & Glomb, 2014). Building on SCT, individuals often assess their competence and status by comparing themselves to others (Festinger, 1954). In organizational contexts, jealousy is more likely to arise when leaders view subordinates with high-leadership potential as social comparison referents (individuals with whom they compare themselves to assess their own competence and status) and perceive them as possessing qualities they lack, which threatens the leader’s self-identity regarding competence and status (H. Li et al., 2024). Leader jealousy triggers a desire to protect one’s status and power, leading to ostracism as a means of weakening the influence of potential threats (Xue et al., 2020).

At the same time, existing research has not reached a consensus on the effects of ELP, possibly due to the existence of certain boundary conditions that cause ELP to have varying impacts. Building on the theoretical framework of SCT, individual emotional responses are often shaped by both the environmental context and individual traits (Jani & Han, 2013; Winter & Kuiper, 1997). In light of this, the present study introduces organizational competitive climate (OCC) and leader’s core self-evaluation (LCSE) as moderating variables to examine their boundary conditions on the effects of ELP in different contexts. Specifically, OCC refers to the perceived atmosphere of competition and stress within an organization, where employees view organizational rewards as a result of comparison with others (Itani & Chaker, 2022; Yıldız et al., 2023). High OCC motivates leaders to focus more on performance, compensation, and status goals, influencing their emotional and behavioral responses toward subordinates (Fousiani & Wisse, 2022; Itani & Chaker, 2022). Under low OCC, however, the motivation to improve performance and outperform subordinates weakens, and leaders’ perceptions of the behaviors they expect from high-potential employees, as well as which behaviors will be rewarded, become more ambiguous, leading to a reduction in negative emotions (David et al., 2021). Core self-evaluation encompasses self-esteem, locus of control, neuroticism, and general self-efficacy, and has been demonstrated to be a stronger predictor of individual behaviors and attitudes than any single personality trait (Judge et al., 2002). Higher LCSE typically exhibit greater self-confidence and a stronger sense of self-worth, making them more likely to adopt positive attitudes rather than rejection behaviors when faced with potential threats (Ferris et al., 2015; Xu et al., 2017). In contrast, leaders with lower core self-evaluation are more sensitive, leading to the development of negative attitudes that hinder positive interactions with subordinates (Chang et al., 2012; Kammeyer-Mueller et al., 2009).

This study advances the existing literature by proposing a theoretically integrative framework that examines how ELP shapes leader ostracism through leader jealousy mechanisms, moderated by OCC and LCSE. Building on these theoretical advancements, we present three key contributions. First, we challenge the prevailing view that ELP always leads to positive outcomes by revealing its dark side—specifically, that ELP can trigger leader ostracism. This finding emphasizes that high-leadership potential employees, while driving organizational growth, may inadvertently become targets of adverse leadership behaviors due to their perceived threat to existing power structures. Second, drawing on SCT, we uncover the emotional mechanism behind ELP’s adverse effects, proposing that leader jealousy mediates this relationship. Leaders’ comparisons to high-leadership potential subordinates can provoke jealousy, which escalates into leader ostracism. Third, we introduce two boundary conditions—OCC and LCSE—and demonstrate their moderating effects on the relationship between ELP and leader ostracism. We find that low OCC reduces the likelihood of leader ostracism, while high LCSE helps leaders manage jealousy constructively, mitigating the negative effects of ELP. These findings enhance our understanding of ELP’s conditional effects and highlight how organizational and individual factors shape leader–subordinate relationships.

## 2. Theoretical Background and Research Hypothesis

### 2.1. Social Comparison Theory

Originally conceptualized by Festinger (1954), SCT posits that, in the absence of objective benchmarks, individuals assess their abilities, perspectives, and social status through comparisons with others. This theory posits that social comparison serves as a critical mechanism for individuals to preserve self-esteem, maintain self-consistency, and adapt to their environment (Wood, 1989). The direction of social comparison can be classified into upward and downward comparisons. The former refers to comparing oneself with others who are perceived to be better in some aspects, which may foster motivation but also evoke negative emotions, such as jealousy and feelings of threat, while the latter refers to comparing oneself with others who are perceived to be worse in certain aspects, which can bolster confidence, albeit potentially fostering complacency and stagnation (Pan et al., 2021). At the same time, social comparisons, while often explicit, can also occur implicitly within an individual’s subjective perceptions, with the propensity to engage in such comparisons exhibiting considerable individual variability (Meier & Johnson, 2022). In organizational settings, social comparison processes are more frequent especially within hierarchically structured, resource-constrained workplaces, where upward and downward comparisons are more likely to occur (Matthews & Kelemen, 2025). Leaders, at the center of organizational power, are often seen as the primary reference point for evaluation criteria (Giessner & Schubert, 2007). Such upward comparisons by subordinates may threaten a leader’s sense of self-worth, triggering defensive emotional responses such as jealousy or hostility, particularly when subordinates exhibit higher-than-usual abilities or potential (M. Li et al., 2023; Pan et al., 2021). Research shows that individuals are more likely to engage in exclusion, denial, or suppression behaviors when they perceive themselves in an unfavorable comparative position to assert superiority (Fernández et al., 2023). ELP, as a highly visible and easily triggered social comparison trait, is likely to catalyze negative social comparisons by superiors (Reh et al., 2022). Leaders may feel threatened and experience jealousy when they recognize that a subordinate possesses exceptional potential, influence, or leadership skills (Mitchell et al., 2024). This emotional reaction not only influences leaders’ attitudes and judgments toward the employee but may also manifest behaviorally through ostracism, resource limitations, and denial of recognition, ultimately affecting the developmental path of high-leadership potential individuals within the organization (Marescaux et al., 2021).

Hence, SCT offers a crucial theoretical framework for this study, suggesting that ELP may encounter developmental barriers due to the negative emotions they evoke in their superiors. Exploring how leaders perceive and respond to ELP within a social comparison context reveals the multifaceted and complex nature of managing high-leadership potential talent in organizations. Building on this theoretical framework, this study investigates how ELP triggers leader jealousy, which subsequently leads to leader ostracism, while identifying key boundary conditions. The aim is to address gaps in the existing literature regarding leaders’ emotional responses and provide theoretical support for constructing a more inclusive and efficacy-oriented talent development system.

### 2.2. Employee’s Leadership Potential and Leader Jealousy

Leadership potential has garnered increasing attention in the fields of organizational behavior and human resource management in recent years (Ghamrawi et al., 2024; Morf & Bakker, 2024). ELP, defined as an employee’s capacity to exhibit strengths in leadership, coordination, problem-solving, and interpersonal influence, serves as a critical reference for organizations in identifying future leaders and implementing succession plans (Troth & Gyetvey, 2014). ELP is typically reflected in three dimensions: (1) Functional Competency—the employee’s excellence in task execution, strategic thinking, and cross-team collaboration; (2) Social Capital—the ability to build high-quality relationships across hierarchical levels within the organization; and (3) Promotion Potential—the identification of the employee as high-leadership-potential talent in the organization’s promotion pipeline, with a focus on cultivating such talent. Existing research has primarily focused on the positive effects of ELP on individuals and organizations, including improvements in team performance, the stimulation of proactive behaviors, and the enhancement of organizational commitment (Steffens et al., 2018). However, ELP can also trigger negative emotional responses from supervisors and create challenges in the leader–subordinate relationship.

Contingent on SCT, individuals choose reference objects for social comparison based on the availability of information in their environment and the similarity of the reference objects (Festinger, 1954; Matthews & Kelemen, 2025). Jealousy is a common emotion that individuals are often hesitant to acknowledge (Parker et al., 2005). Leader jealousy arises when leaders feel envious of certain advantages, such as abilities, achievements, or resources, that others possess but they lack (Braun et al., 2018). Smith and Kim (2007) argued that jealousy is a complex emotion that emerges when an individual compares themselves to others and perceives themselves to be in a disadvantaged position, encompassing feelings such as anger, inferiority, hostility, and pain. Leaders continually validate their self-perceptions and social positioning in the workplace by comparing themselves to their subordinates (H. Liu et al., 2021). Subordinates may become the focus of upward comparisons by leaders when they demonstrate exceptional influence, performance, or developmental potential, even surpassing their current superiors (Gerpott & Van Quaquebeke, 2023). As ELP disrupts role stability, it can trigger a leader’s sense of self-threat, leading to insecurity, jealousy, and even hostility (Xie et al., 2023; Xue et al., 2020). Within this theoretical framework, this study argues that ELP triggers leader jealousy in three key ways (Duffy et al., 2012; Feather, 2015). (1) Shaky self-identification of competence. In organizations, leaders’ recognition of their competence typically hinges on professional judgment, task control ability, and accumulated experience (Dragoni et al., 2011). When subordinates exhibit leadership potential, such as strategic thinking and high executive ability, it can challenge the leader’s perception of their own competence, prompting the leader to compare themselves to these subordinates. If the leader perceives that they are losing their comparative advantage in terms of professionalism or leadership judgment, their sense of self-identity may be destabilized (Kets de Vries, 1994). Consequently, if leaders cannot restore psychological balance by reaffirming their value or devaluing the subordinate, jealousy may emerge as a response to the perceived threat to their self-esteem (C. Liu et al., 2021). (2) The stimulation of positional insecurity. Subordinates with leadership potential are often recognized as strategic talents by the organization, gaining access to more development resources and promotion opportunities. Leaders may perceive a threat to the stability of their positions if they sense that their promotion paths are becoming clearer or their network resources are expanding (Wisse et al., 2019). When leaders are unable to alleviate this positional uncertainty by reasserting authority or managing boundaries, positional anxiety is internalized as jealousy in response to situations such as resource threats and positional inequality. (3) Weakened sense of power control. A leader’s control over the team is derived not only from formal positional power but also from their informal position in managing information flow, task allocation, and interpersonal interactions in daily operations (Oedzes et al., 2019). However, as ELP becomes more prominent and their informal influence solidifies within the team, leaders may perceive a loss of their original control (Luria & Berson, 2013). This is especially true when team members begin to align more with the views of the high-leadership-potential subordinate, or when team resource allocation and decision-making increasingly rely on the subordinate’s coordination, causing the leader to feel a loss of control over their sphere of influence, which can trigger jealousy. Accordingly, this study proposed the following hypothesis:

**H1.**  *Employee* *’s leadership potential positively influences leader jealousy.*

### 2.3. The Mediating Role of Leader Jealousy

Furthermore, according to SCT, maintaining a positive sense of self-worth is a fundamental human need. When individuals encounter negative social comparison information, they adopt various coping mechanisms to mitigate the perceived threat (Caliskan et al., 2024). Based on this, this study suggests that ELP leads to leader ostracism by increasing leader jealousy. Workplace ostracism is a typical form of socially harmful behavior that often manifests in more subtle ways, such as neglect, coldness, avoidance of communication, or disregard for emotional needs (Konieczna, 2024; Ren et al., 2018). In contrast to verbal aggression or overt confrontation, ostracism is a more strategic and sustainable approach to isolating or excluding an individual or organization (Pfundmair et al., 2024). Previous research on workplace ostracism has primarily focused on the effects experienced by the individuals who are ostracized. This study, however, examines the relationship between leader jealousy and active ostracism behaviors from the perspective of the excluder (leader), specifically in relation to subordinates.

In conjunction with the definition of jealousy as a negative social comparison process, which outlines the conditions under which ostracism may occur, the characteristics of high-leadership potential employees make them particularly susceptible to ostracism. The primary motivation of individuals experiencing jealousy is to alleviate cognitive dissonance by reconciling their position with that of the object of their jealousy (Khan et al., 2014). Jealousy causes leaders to scrutinize high-leadership potential employees more closely, and this hostility creates tension and disgust, triggering negative perceptions of these employees, which can harm both their work and personal lives (van Zelderen et al., 2023; Xue et al., 2020). Considering the existing literature, this study finds that leaders driven by jealousy may actively marginalize high-leadership potential employees to achieve three primary objectives: first, to alleviate the psychological discomfort caused by perceived inferiority in ability or status, thereby maintaining their self-esteem (Khan et al., 2014); second, to weaken the subordinate’s organizational influence and upward momentum by cutting off interpersonal solidarity and access to information, thus narrowing the gap between them (Duffy et al., 2012); and third, to gain emotional compensation through hurtful behaviors, balancing the negative emotions generated by low self-esteem (Bilal et al., 2021). As discussed above, leader jealousy is a psychological reaction with clear behavioral directionality, and the resulting leader ostracism not only reflects a defensive strategy but also acts as a key mediator through which ELP influences leadership behavior. Therefore, we proposed the following hypothesis:

**H2.**  *Leader jealousy mediates the relationship between employee* *’s leadership potential and leader ostracism.*

### 2.4. The Moderating Role of Organizational Competitive Climate

OCC refers to the organizational climate in which employees’ perceptions of competitiveness and stress arise from comparing themselves with others in terms of performance, compensation, status, and rights (Itani & Chaker, 2022; Yıldız et al., 2023). It reflects individuals’ perceptions of the extent to which they compete with other members of the organization (Zhang, 2025). Leaders, being at the center of the team, are particularly sensitive to personal gains and losses (Brown et al., 1998; Wisse et al., 2019). It has been noted that OCC reinforces individuals’ social comparison processes, influencing their cognitive, emotional, and behavioral responses (Fletcher et al., 2008). For leaders with decision-making and voice power in an organization, high OCC means they are more likely to focus on the power and competence gaps between themselves and their subordinates. This is particularly true when faced with employees with high-leadership potential, whom leaders may perceive as a potential threat, triggering negative consequences such as jealousy and rejection (Murtza & Rasheed, 2023; Ng, 2017).

Based on SCT, individuals tend to assess their competence and performance by making upward social comparisons with those who possess similar but dominant traits, particularly in status-neighboring or resource-constrained contexts (M. Li et al., 2023; Reh et al., 2022). Specifically, when OCC is high, organizational members are more likely to engage in frequent and in-depth social comparisons with their colleagues (Yıldız et al., 2023). In this highly competitive environment, leaders are inevitably involved in social comparisons with their subordinates, particularly those with higher ELP. Brown et al. (1998) clarified that, in high OCC situations, individuals are more likely to be acutely aware of factors such as others’ performance, achievements, and potential, leading them to engage more frequently in upward comparisons that make them feel inferior. Moreover, such frequent comparisons not only make leaders more acutely aware of their shortcomings in certain areas but also intensify their psychological unease and sense of threat (Wisse et al., 2019). For example, when leaders observe that their subordinates demonstrate leadership traits such as communication, decision-making, and influence that surpass their own, they are more likely to perceive these subordinates with high ELPs as a potential competitive threat, or even as direct competitors for their position and resources, triggering stronger feelings of jealousy (Vecchio, 2000; Tai et al., 2012). This emotion intensifies the leader’s negative self-perception of their own competence, ultimately exacerbating psychological stress and reinforcing negative emotions, including jealousy. In contrast, when OCC is low, the intensity of social comparison among organizational members significantly decreases. In such an environment, leaders are less focused on differences in performance, potential, and resource allocation among members, and as a result, they perceive the competence gap between themselves and their high ELP subordinates less frequently. Previous research has shown that low-competitive environments foster a more tolerant and supportive organizational climate, where leaders are more likely to positively acknowledge and recognize subordinates’ abilities and potential rather than viewing them as threats or competitors (Lee et al., 2025; Reh et al., 2018). Additionally, lower competitive pressures lead leaders to base their evaluations of self-worth and competence more on internal criteria than on external comparisons, which significantly reduces jealousy induced by negative social comparisons (comparisons in which individuals perceive themselves as inferior to others, often leading to feelings of jealousy and insecurity). For example, Watkins (2021) noted that low OCC effectively reduces the generation and accumulation of negative leader emotions by mitigating contrastive evaluations and the sense of competition, making it less likely to trigger strong leader jealousy. Stated formally, we hypothesize the following:

**H3.**  *Organizational competitive climate moderates the relationship between employee’s leadership potential and leader jealousy.*

Further, this study proposes that OCC not only moderates the direct relationship between ELP and leader jealousy but also influences the mediating effect of leader jealousy between ELP and leader ostracism. According to SCT, individuals who perceive a gap between themselves and their comparators in terms of key competencies or resources are likely to react behaviorally in order to reduce their internal feelings of discomfort and threat (Greenberg et al., 2007; Meier & Johnson, 2022). When OCC is high, leaders exhibit heightened sensitivity to competency gaps and competition for resources in competitive environments, which makes leader jealousy toward high-leadership potential employees more likely to be triggered and sustained. This jealousy, in turn, drives leaders to adopt negative behaviors, such as ostracizing or marginalizing these high-leadership potential employees, in response to perceived threats (Duffy et al., 2012; E. Kim & Glomb, 2014). Specifically, high OCC amplifies threat perceptions among leaders, leading them to view high-leadership potential employees as direct challengers to their authority and resource security. In such contexts, leader jealousy often manifests as leader ostracism, further strengthening its mediating role in influencing leader behavior (Schaubroeck & Lam, 2004). In contrast, when OCC is low, the intensity of social comparisons and competitive perceptions among organizational members is significantly reduced. In this environment, while leaders may perceive a competence advantage in high-leadership potential employees, this perception is insufficient to generate sustained feelings of threat or significant negative emotions. Leaders are more likely to engage with their subordinates in positive and proactive ways rather than resorting to negative behavioral coping strategies (Ng, 2017). Additionally, low OCC fosters more opportunities for collaboration and interaction between leaders and employees, encouraging leaders to respond with a positive mindset, which in turn highlights their leadership potential. This dynamic effectively reduces the accumulation of leader jealousy and the resulting leader ostracism (Mohd Shamsudin et al., 2024; Wang & Sung, 2016). In line with these observations, this study postulates the following:

**H4.**  *Organizational* *competitive climate moderates the mediating role of leader jealousy between employee’s leadership potential and leader ostracism.*

### 2.5. The Moderating Role of Leader’s Core Self-Evaluation

As a fundamental evaluation of individuals’ abilities and values, core self-evaluation is characterized by stability and strong representativeness and is considered a form of personality trait expression (Bono & Judge, 2003; Raza et al., 2023). High core self-evaluation is characterized by adaptability, positivity, confidence, and a belief in one’s ability to control their life (Ramaprasad et al., 2022). Most existing literature focuses on employees’ core self-evaluation, with less attention given to the effects of LCSE on subordinates. Ahn et al. (2018), drawing on social exchange theory, found that LCSE can enhance employees’ job performance by improving ethical leadership; Hu et al. (2012) verified LCSE’s positive impact on followers’ perceptions of transformational leadership. Given this, this study attempts to introduce LCSE to explore the differences in the impact of ELP on leader ostracism, specifically whether variations in LCSE lead to significantly different outcomes.

SCT emphasizes that individuals form self-evaluations through comparisons with others, and core self-evaluation, as a stable aspect of self-perception, influences their sensitivity and response patterns in social comparisons (Caliskan et al., 2024; Chang et al., 2012). Leaders with high core self-evaluation tend to possess greater self-confidence, a stronger sense of control, organizational identity, and are more likely to affirm their abilities and value. Even when faced with subordinates who display high ELP, they rationally assess the competence gap and are less prone to feelings of threat or negative emotions (Ahn et al., 2018). This aligns with the findings of Simsek et al. (2010) and others, who suggest that when LCSE is high, individuals are more confident in their ability to control their environment and are thus more inclined to pursue potential advantages in uncertain opportunities. Joo et al. (2010) further pointed out that individuals with high core self-evaluation evaluate themselves more positively and proactively, are more willing to attribute meaning and value to their work, and maintain stable self-perceptions and an optimistic outlook. Meanwhile, Sun et al. (2023) found that individuals with a positive and proactive core self-evaluation are less likely to adjust their motivation and behavior in response to external circumstances and information. Therefore, in the process of social comparison, when LCSE levels are high, even in the face of competence threats posed by high ELP subordinates, their self-perceptions and emotional responses remain relatively stable and positive, making them less prone to strong feelings of jealousy. Conversely, when LCSE levels are low, individuals’ self-perceptions and beliefs in their competence are relatively fragile, making them more susceptible to external pressures during social comparisons (Judge et al., 1998). When faced with high-leadership potential subordinates, these leaders often lack sufficient self-confidence, leading them to overestimate others’ strengths and underestimate their value, which triggers self-doubt and negative emotions (Chang et al., 2012). This emotional response further amplifies the effect of high ELP on leader jealousy. Furthermore, when LCSE is low, leaders’ self-perception stability and competence beliefs are weaker, making them more susceptible to external influences during social comparisons (Judge et al., 1998). In the face of high ELP subordinates, such leaders tend to lack positive recognition of their competence and are more inclined to perceive a competence gap with their subordinates, thereby increasing the likelihood of negative emotional experiences (Chang et al., 2012). In this context, the role of ELP in stimulating leader jealousy is more pronounced. Hence, the following hypothesis is formulated:

**H5.**  *Leader’s core self-evaluation moderates the relationship between employee’s leadership potential and leader jealousy.*

In addition to this, LCSE affects leaders’ emotional responses to social comparisons and moderates how these emotions are expressed through specific behaviors. Whether and how leaders externalize jealousy, triggered by high ELP subordinates, into rejection behaviors depends on the strength and stability of their self-perceptions. When LCSE is high, leaders typically possess more mature emotional regulation and stronger psychological resources. Even when jealousy is aroused, they are more likely to adjust through rational thinking, constructive dialogue, or self-improvement, thus diminishing the effect of jealousy transforming into rejection behavior (Ahn et al., 2018; Joo et al., 2010). However, lower LCSE indicates a lack of effective emotion regulation, making individuals more susceptible to emotionally driven, impulsive, or defensive behavioral responses, such as alleviating feelings of discomfort and threat by ostracizing high ELP subordinates (Ferris et al., 2008). Therefore, differences in LCSE levels determine the intensity with which leader jealousy is translated into actual leader ostracism. In summary, high LCSE attenuates the mediating effect of leader jealousy between ELP and leader ostracism behaviors, while low LCSE reinforces this adverse transduction effect. Combined with the above arguments, we propose the following hypothesis:

**H6.**  *Leader’s core self-evaluation moderates the mediating role of leader jealousy between employee’s leadership potential and leader ostracism.*

**Figure 1:**
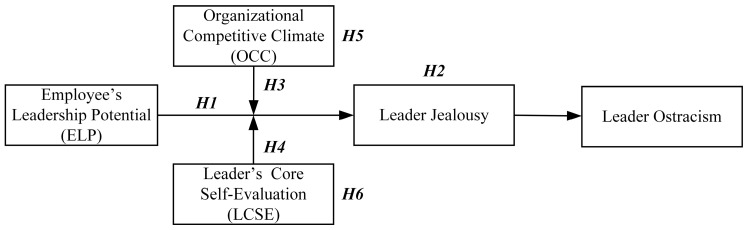
Theoretical model.

The theoretical model presented in this study, illustrated in Figure 1, builds upon previous work using two-moderator frameworks (Guo et al., 2019; Ng et al., 2022). It positions ELP as the independent variable and leader ostracism as the dependent variable, with leader jealousy acting as a mediator. Consistent with research on conditional mechanisms, OCC and LCSE are introduced as moderators of the relationship between ELP and leader jealousy. This approach aligns with established practices in organizational behavior research involving moderated mediation models.

## 3. Methods

### 3.1. Sample and Procedures

The data for this study were collected through field research across three large state-owned construction companies in central China. The sample is characterized by the prevalence of a project team system in the construction industry, where there are closer interactions between leaders and subordinates, as well as greater dependence and collaboration among team members. This dynamic makes the impact of leader exclusionary behaviors on team climate and job performance particularly significant (Demirkesen & Tezel, 2022; Yang et al., 2011). First, to ensure the reliability of the findings and uphold the principle of confidentiality, several consultations and discussions were held with the heads of the human resources departments of the companies before conducting the formal research, thereby ensuring the smooth progress of the study. Second, data collection was conducted on-site, with structured interviews and questionnaires administered by trained research assistants to ensure consistency and reliability in the data collection process. To minimize the effects of homogeneity bias and causality lag, data were collected in three stages, with a one-month time interval between each stage. Additionally, the entire process was strictly coded. In the first phase, 743 questionnaires were distributed, and 719 were returned. These were completed by leaders who provided individual demographic characteristics such as gender, age, education, and tenure, and by team leaders who assessed their core self-evaluation and the extent of the OCC. In the second stage, team leaders evaluated the leadership potential of their subordinates and their level of jealousy. A total of 719 questionnaires were distributed, and 698 were returned. In the third stage, leaders were asked to complete a questionnaire on their ostracism of subordinates. A total of 698 questionnaires were distributed, and 672 were returned in this stage. The overall effective recovery rate across all three stages was 90.44%, calculated by excluding incomplete or invalid responses such as those with missing data or inconsistent answers. Among the participants, 85.9% are men and 14.1% are women. Regarding education level, 1.5% have completed high school or lower, 19.6% have completed specialized education, 73.7% hold undergraduate degrees, and 5.2% have a master’s degree or higher. In terms of age distribution, 0.7% are between 21–25 years old, 26.9% are between 26–30 years old, 57.4% are between 31–35 years old, 8.8% are between 36–40 years old, and 6.1% are 41 years old or older. Regarding tenure, 1.8% have 5 years or less, 14.6% have 6–10 years, 40.5% have 11–15 years, 33.3% have 16–20 years, and 9.8% have 21 years or more.

### 3.2. Measures

This study adhered strictly to the standard procedure of translating and back-translating established, validated scales. To ensure the accuracy of the Chinese version, three professors in business administration and several doctoral students were invited to evaluate the scale against the original version. After making reasonable adjustments and optimizing certain items, the reliability and validity of the Chinese scales were found to be satisfactory. All scales were rated on a 5-point Likert scale.

Employee’s leadership potential (T2): The scale developed by Mueller et al. (2011) was used, which includes four items, such as “I think he/she has the skills to lead’ and ‘I think he/she can rise to a leadership position.” The Cronbach’s α for this scale was 0.918.

Organizational competitive climate (T1): The Brown et al. (1998) scale was used, which contains four questions, such as “Within the organization, my leaders often compare my performance with that of other colleagues” and “In the organization, everyone wants to be the best performer”. The Cronbach’s α for this scale was 0.899.

Leader core self-evaluation (T1): The scale used was that of Judge et al. (2003), which consists of 12 items, such as “I believe that I can achieve what I want to achieve in life” and “If I try hard enough, I usually succeed”. The Cronbach’s α for this scale was 0.859.

Leader jealousy (T2): The scale used was E. Kim and Glomb’s (2014), which includes four items, such as “It is frustrating to see this person succeed so easily” and “I usually feel inferior about this person’s success”. The Cronbach’s α for this scale was 0.929.

Leader ostracism (T3): Ferris et al.’s (2008) scale was used, which includes six items, such as “Ignores or fails to respond to your communication” and “Doesn’t allow you to participate in important work events or meetings”. The Cronbach’s α for this scale was 0.889.

Controlled variables (T1): Based on published research on jealousy and ostracism (Bani-Melhem et al., 2023; Jawahar et al., 2021), this study used the participants’ demographic information as control variables, including the leader’s gender, education, age, and tenure.

## 4. Results

### 4.1. Common Method Biases

To mitigate potential common method bias (CMB), this study implemented a three-wave data collection design and assured participants of confidentiality to reduce evaluation apprehension and temporal consistency artifacts (Podsakoff et al., 2003). As an initial diagnostic, Harman’s single-factor test revealed that the first factor accounted for 32.48% of the total variance, below the commonly accepted threshold of 40%, suggesting CMB was unlikely to be a significant concern. Additionally, we employed the unmeasured latent method factor technique by integrating a method factor into the hypothesized five-factor measurement model. The resulting six-factor model demonstrated only marginal improvement in fit (∆CFI = 0.007, ∆TLI = 0.002, ∆RMSEA = 0.014), indicating that the inclusion of the method factor did not substantially enhance model fit (Richardson et al., 2009). Collectively, these results suggest that CMB is unlikely to distort the study’s findings meaningfully.

### 4.2. Confirmatory Factor Analysis

The confirmatory factor analysis results using Mplus 8.3 are shown in Table 1. The five-factor model provides the best fit compared to other factor models, indicating good discriminant validity. The five-factor model (χ^2^/df = 3.566, CFI = 0.926, TLI = 0.918, SRMR = 0.048, RMSEA = 0.062) fits the data well. In contrast, the four-factor model (χ^2^/df = 7.767, CFI = 0.803, TLI = 0.785, SRMR = 0.105, RMSEA = 0.100) shows poor fit.

### 4.3. Descriptive Analysis

Descriptive statistics and correlation analysis were conducted using SPSS 27.0. As shown in Table 2, the ELP was positively correlated with leader ostracism (r = 0.37, *p* < 0.01) and leader jealousy (r = 0.35, *p* < 0.01). Leader jealousy was positively correlated with leader ostracism (r = 0.48, *p* < 0.01). This provides preliminary empirical support for the theoretical model of this study.

### 4.4. Hypotheses’ Tests

#### 4.4.1. Mediating Effect Test

This study used the SPSS 27.0 and employed the bootstrap method with 5000 repetitions to test the mediating effects. The regression analysis results are shown in Table 3. First, the results of Model 2 and Model 4 indicate that, after controlling for demographic variables, ELP significantly positively predicts leader jealousy (β = 0.333, *p* < 0.01) and leader ostracism (β = 0.362, *p* < 0.01). Thus, H1 was verified. Second, Model 5 indicates that leader jealousy had a significant positive effect on leader ostracism (β = 0.240, *p* < 0.01). Therefore, H2 was supported.

Furthermore, as shown in Table 4, the total effect of the ELP on leader ostracism was significant because the 95% confidence interval excluded 0. The direct effect of ELP on leader ostracism was significant because the 95% confidence interval excluded 0. The ELP had a significant positive effect on leader ostracism through leader jealousy, and its 95% confidence interval did not contain 0, suggesting that leader jealousy played a partial mediating role. Thus, H2 was further verified.

#### 4.4.2. Moderating Effect Test

This study used the SPSS 27.0 to construct the hierarchical regression analysis to empirically test their moderating effects. The results are presented in Table 5. The results of Model 8 showed that the interaction of ELP and OCC had a significant positive effect on leader jealousy (β = 0.217, *p* < 0.01), indicating that OCC can positively moderate the effect of ELP on leader jealousy. Thus, H3 was supported. Additionally, the results of Model 10 showed that the interaction of ELP and LCSE had a significant negative effect on leader jealousy (β = −0.527, *p* < 0.01), indicating that LCSE can negatively moderate the effect of ELP on leader jealousy. Therefore, H5 was supported.

**Figure 2:**
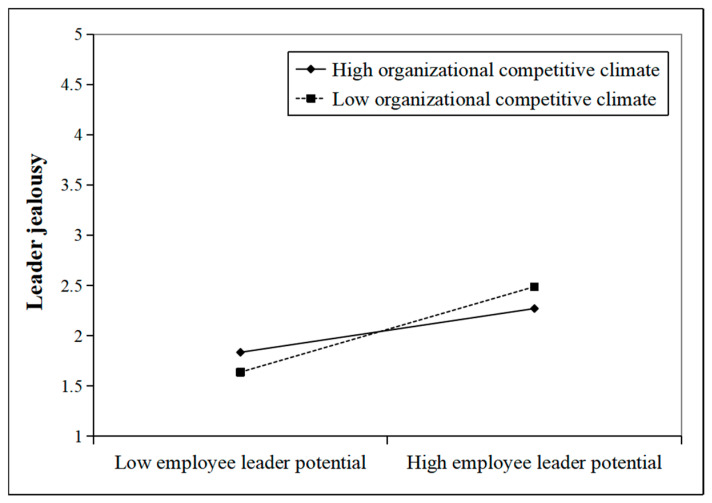
Moderating role of OCC.

**Figure 3:**
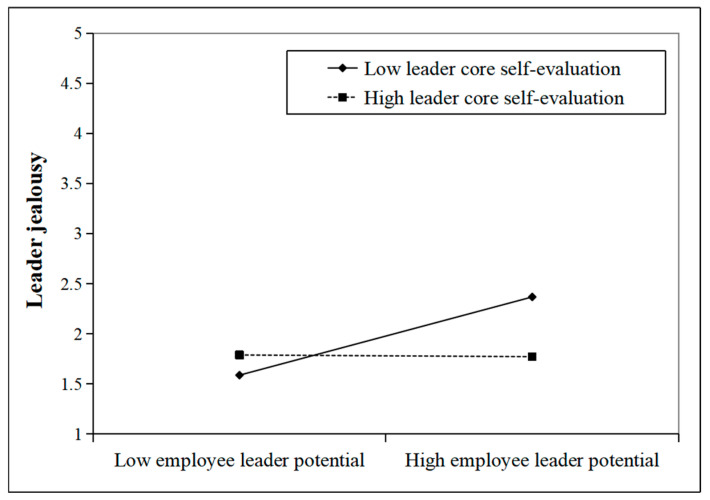
Moderating role of LCSE.

To further illustrate the moderating effects of OCC and LCSE on the relationship between ELP and leader jealousy, this study used the simple slope analysis method to draw the moderating effect diagrams with above and below the mean by one standard deviation of OCC and LCSE, respectively. As shown in Figure 2, the positive effect of ELP on leader jealousy was stronger under higher OCC. Figure 3 shows that the negative effect of ELP on leader jealousy was weaker when LCSE was higher. As a result, H3 and H5 were further validated.

#### 4.4.3. Moderated Mediating Effect Test

Bootstrap analysis was conducted in the PROCESS to test the moderated mediating effect further. The results are presented in Table 6. It shows that if the OCC was low, then the effect was 0.096 (95% bias-corrected CI = [0.023, 0.166]), which did not include 0, and if the OCC was high, then the effect was 0.188(95% bias-corrected CI = [0.083, 0.203]), which did not include 0. Moreover, the effect of the differences between the high and low levels was 0.092 (95% bias-corrected CI = [0.007, 0.201]), which did not include 0. The moderated mediating effect of the OOC was significant. Therefore, H4 was validated. Similarly, if the LCSE was low, then the effect was 0.173 (95% bias-corrected CI = [0.118, 0.232]), which did not include 0, and if the LCSE was high, then the effect was −0.004 (95% bias-corrected CI = [−0.055, 0.044]), which included 0. Lastly, the effect of the differences between the high and low levels was -0.177 (95% bias-corrected CI = [−0.241, −0.121]). This proves that the moderated mediating effect of the LCSE was significant. Therefore, H6 was supported.

**Figure 4:**
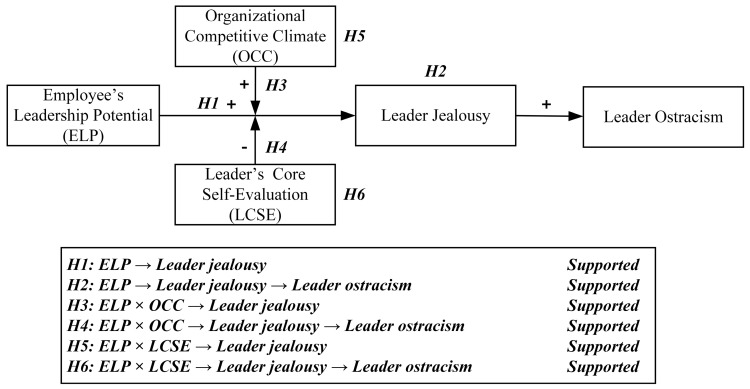
Summary of hypotheses and empirical results.

As Figure 4 shows, the conceptual model illustrates the relationships between ELP, leader jealousy, and leader ostracism, with OCC and LCSE serving as moderators. The results of hypothesis testing are summarized, indicating support for all proposed relationships.

## 5. Discussion

Based on SCT, this study aims to bridge the existing research gap by uncovering the underlying mechanism between ELP and leader ostracism. It also establishes a comprehensive theoretical model to interpret this process, considering both individual leader traits and the organizational context. The following conclusions were drawn.

Firstly, this study highlights the effect of ELP on leader ostracism, revealing the mediating role of leader jealousy. Individuals with high ELP trigger leader jealousy through superior competencies, leading to negative emotional responses and, in turn, to leader ostracism. This supports SCT, which suggests jealousy arises when individuals perceive others as surpassing them in key areas, threatening their self-worth and status (Greenberg et al., 2007; Meier & Johnson, 2022). Contrary to the view that high ELP always leads to positive outcomes, this study shows that when high-leadership potential employees are perceived as a threat, it triggers negative responses, including leader ostracism (Graves & Sarkis, 2018; Othman & Mahmood, 2022). This finding provides a more integrative perspective on the effects of ELP, showing that its impact depends on how it activates psychological mechanisms such as leader jealousy.

Secondly, OCC moderates the relationship between ELP and leader jealousy. In highly competitive environments, leaders are more likely to see high-leadership potential employees as rivals, intensifying the perception of threat and jealousy (Murtza & Rasheed, 2023; Xue et al., 2020). In contrast, in low-competitive environments, leaders may be more supportive and less likely to view high-leadership potential employees as threats, reducing jealousy (Lee et al., 2025; Reh et al., 2018). These findings underscore the role of organizational context in shaping the perception of ELP.

Thirdly, LCSE moderates the relationship between ELP and leader jealousy. Leaders with high core self-evaluation are more confident in their abilities and less likely to perceive ELP as a threat, minimizing jealousy (Chang et al., 2012). Conversely, low core self-evaluation leaders may feel inadequate when facing high-leadership potential employees, leading to heightened jealousy and leader ostracism (Sun et al., 2023; Judge et al., 2003). This finding aligns with the concept of LCSE, which suggests that individuals with higher self-worth are less likely to feel threatened by others’ success, reducing jealousy (Bono & Judge, 2003).

### 5.1. Theoretical Implications

There are several theoretical contributions to this paper. First, this study uncovers the dark side of ELP, challenging the widely held belief that ELP always leads to positive outcomes for organizations. While prior research has primarily focused on the positive aspects of high ELP—such as fostering innovation and driving organizational growth—this study reveals that high ELP can, in fact, trigger leader ostracism (DiLiello & Houghton, 2006; Troth & Gyetvey, 2014). High-leadership potential employees, despite their contributions to organizational success, may inadvertently provoke negative leadership responses as their perceived threat to existing power structures leads to relational tensions and exclusionary behaviors from leaders (D. Liu et al., 2012; Mueller et al., 2011). This contribution expands the current understanding of ELP by demonstrating that its effects are not always beneficial and can result in unintended negative consequences within leader–subordinate relationships.

Second, we build upon SCT to identify the emotional underpinnings of ELP’s negative consequences. Specifically, we propose that leader jealousy serves as a mediator between ELP and leader ostracism. Our findings show that leaders who perceive subordinates with high-leadership potential as competitors or threats are likely to experience jealousy, which can escalate into ostracism (Xue et al., 2020; Wang & Sung, 2016). This mediating role of leader jealousy highlights the emotional dynamics that shape leader–subordinate relationships, providing a deeper understanding of the psychological processes that contribute to negative leader behaviors in the workplace. Additionally, this study was conducted within the Chinese cultural context, characterized by a strong emphasis on hierarchical structures and authority, which shape leader behaviors such as jealousy and ostracism (Westwood, 1997). Notwithstanding these cultural specifics, the fundamental dynamics between leadership potential, leader jealousy, and leader ostracism hold cross-cultural relevance. The psychological processes underlying social comparison and interpersonal dynamics—key drivers of jealousy and ostracism—are universal, even though their manifestations may vary across different cultural settings (Mohd Shamsudin et al., 2024; Tai et al., 2012).

Third, we extend SCT to leadership and management by introducing two critical moderators—OCC and LCSE—to explore how contextual and individual factors influence the relationship between ELP and leader ostracism. Our results show that a high OCC exacerbates the relationship between ELP and leader jealousy, intensifying the negative emotional response (Bani-Melhem et al., 2023; Murtza & Rasheed, 2023). On the other hand, a high LCSE allows leaders to manage feelings of jealousy better, thereby mitigating the likelihood of leader ostracism (H. Kim & Jang, 2023; Mohd Shamsudin et al., 2024). These findings add depth to SCT by demonstrating how both the organizational environment and individual leader traits can influence emotional reactions and leadership behavior, offering a more comprehensive framework for understanding the complex interplay between employee potential and leader behaviors.

### 5.2. Practical Implications

First, organizations should develop strategies for managing high ELP that account for the risk of leader jealousy and leader ostracism. Given the significant mediating role of leader jealousy in the relationship between ELP and leader ostracism, companies should be proactive in creating an environment where leadership potential is recognized and nurtured without triggering negative emotions among leaders (Geddes et al., 2020; Yu et al., 2018). To do so, organizations can implement leadership development programs that not only highlight the strengths of high-potential employees but also ensure that their presence does not feel threatening to current leadership. Leaders can benefit from training programs that promote emotional intelligence, helping them recognize and manage their feelings of jealousy and competitive tension (Walter et al., 2012). Additionally, introducing feedback mechanisms where leaders and employees can discuss potential conflicts and perceptions of competition can help reduce the likelihood of leader ostracism (Chang et al., 2012).

Second, fostering a healthy OCC is essential to mitigating the negative consequences of ELP. Research shows that a high OCC can exacerbate leader jealousy and leader ostracism. To manage this, companies should cultivate a more collaborative, supportive, and less competitive atmosphere. For employees, organizations should create clear career development paths that emphasize collaboration over competition. For managers, organizations can introduce leadership coaching programs that focus on fostering inclusivity and promoting a growth-oriented culture, where employees’ success is celebrated rather than perceived as a threat (Warren et al., 2019). On an organizational level, it is recommended that companies regularly assess their competitive climate through employee surveys and adjust their strategies to ensure a more balanced and supportive work environment (Sharma & Dhar, 2022).

Third, organizations should consider the role of LCSE when designing talent management strategies. Leaders with higher LCSE are less likely to experience jealousy in the face of high-leadership potential employees, suggesting that investing in the self-esteem and psychological well-being of leaders is critical (Gardner, 2020). To achieve this, companies should integrate LCSE assessments into their leadership development programs, offering support to leaders who may have lower self-evaluations and may be more susceptible to feelings of insecurity when confronted with high-leadership potential employees (Day et al., 2021). Tailored mentorship and coaching initiatives that focus on building confidence, resilience, and emotional regulation can help reduce the likelihood of leaders turning to ostracism as a defensive reaction (Al-Romeedy et al., 2025). At the organizational level, it is beneficial to create a culture of psychological safety where leaders feel empowered to embrace their team’s potential without feeling threatened (Perrmann-Graham et al., 2022).

### 5.3. Limitations and Future Directions

This study has several limitations that provide avenues for future research. First, variables were measured using leader self-reports, and variables like leader ostracism are subject to strong social approval effects, making CMB inevitable. Although we minimized respondents’ sense of ambiguity and uncertainty by implementing both ex ante controls and ex post assessments, along with providing detailed explanations for each item, some bias may remain. To mitigate common method bias in future studies, we recommend using scenario-based approaches or experimental designs that indirectly measure these variables by observing leaders’ behaviors and attitudes toward employees with high-leadership potential.

Second, drawing on SCT, this study primarily examines leader jealousy as a mediating mechanism. However, it is important to acknowledge that ELP could also influence leaders’ attitudes and behaviors through alternative psychological pathways. For instance, according to role theory, leaders navigate multiple roles within an organization, and ELP may affect their behavioral outcomes by shaping their perceptions and expectations associated with these roles (Anglin et al., 2022). Additionally, self-determination theory posits that individuals experience heightened motivation and engage in more positive behaviors when their intrinsic needs for autonomy, competence, and relatedness are fulfilled (Kanat-Maymon et al., 2020). ELP may thus enhance a leader’s intrinsic motivation, fostering greater support for and commitment to the team. In light of these perspectives, future research should explore how ELP influences leader behavior through these other psychological mechanisms, offering a broader and more nuanced explanatory framework.

Third, industry characteristics may influence the effect of the findings of this study. Given that our research sample is confined to the construction industry, the extent to which these results can be generalized to other sectors remains uncertain. Therefore, the external validity and broader applicability of these findings warrant further investigation. Additionally, there is potential for further refinement in the methodology for quantitatively excluding leaders who do not engage in social comparisons with employees demonstrating high-leadership potential. Future studies could employ situational simulations or experimental designs to observe leaders’ responses in contrast to high-leadership potential employees, thereby providing a more robust approach to isolating this effect.

## Figures and Tables

**Table 1:** Confirmatory factor analysis of model fit.

Model	Factors	χ^2^/df	χ^2^	df	CFI	TLI	SRMR	RMSEA
Five-factor model	ELP; OCC; LCSE; LJ; LO	3.566	1408.571	395	0.926	0.918	0.048	0.062
Four-factor model	ELP + OCC; LCSE; LJ; LO	7.767	3098.919	399	0.803	0.785	0.105	0.100
Three-factor model	ELP + OCC + LCSE; LJ; LO	11.075	4452.164	402	0.704	0.680	0.107	0.122
Two-factor model	ELP + OCC + LCSE + LJ; LO	15.756	6365.468	404	0.565	0.531	0.128	0.148
One-factor model	ELP + OCC + LCSE + LJ + LO	28.330	12,210.229	431	0.140	0.132	0.304	0.202

Notes: N = 672; ELP = Employee’s leadership potential; LJ = Leader jealousy; OCC = Organizational competitive climate; LCSE = Leader’s core self-evaluation; LO = Leader ostracism.

**Table 2:** Descriptive analysis of all variables.

Variable	M	SD	1	2	3	4	5	6	7	8
1. Gender	1.14	0.35								
2. Education	2.83	0.53	0.05							
3. Age	2.93	0.79	−0.01	−0.32 **						
4. Tenure	3.35	0.91	−0.07	−0.23 **	0.69 **					
5. ELP	2.12	0.76	0.12 **	−0.02	0.01	−0.09 *				
6. Leader jealousy	1.77	0.96	0.09 *	−0.04	0.01	−0.12 **	0.35 **			
7. OCC	1.96	0.63	0.08 *	−0.01	−0.02	−0.04	0.14 **	0.04		
8. LCSE	3.98	0.50	−0.13 **	−0.03	0.10 **	0.12 **	−0.38 **	−0.34 **	−0.34 **	
9. Leader ostracism	2.63	0.88	0.05	0.01	−0.01	−0.06	0.37 **	0.48 **	0.11 **	−0.43 **

Notes: * *p* < 0.05; ** *p* < 0.01; N = 672.

**Table 3:** The results of hierarchical regression model.

Variable	Leader Jealousy		Leader Ostracism		
	Model 1	Model 2	Model 3	Model 4	Model 5
Gender	0.076 *	0.038	0.040	−0.001	−0.015
Education	−0.047	−0.039	0.008	0.017	0.031
Age	0.138 *	0.098	0.063	0.018	−0.018
Tenure	−0.219 **	−0.160 **	−0.096	−0.032	0.027
ELP		0.333 **		0.362 **	0.240 **
Leader jealousy					0.368 **
R2	0.035	0.142	0.007	0.134	0.243
F	5.963 **	21.966 **	1.170	20.618 **	36.951 **

Notes: * *p* < 0.05; ** *p* < 0.01; N = 672.

**Table 4:** Bootstrapping mediation testing results.

Pathway	Effect	SE	t	95% CI	
				Low	High
Total effect	0.420	0.043	9.886	0.337	0.504
Direct effect	0.278	0.042	6.629	0.196	0.361
ELP → Leader jealousy → Leader ostracism	0.142	0.032	0.032	0.083	0.209

**Table 5:** The results of the moderation effects.

Variable	Leader Jealousy				
	Model 6	Model 7	Model 8	Model 9	Model 10
Gender	0.076	0.039	0.087	0.020	−0.021
Education	−0.047	−0.039	−0.072	−0.041	−0.033
Age	0.138 *	0.097	0.121	0.116 *	0.171 *
Tenure	−0.219 *	−0.161 **	−0.166 **	−0.154 *	−0.172 **
ELP		0.334 **	0.423 **	0.247 **	0.251 **
OCC		−0.013	0.008		
ELP × OCC			0.217 **		
LCSE				−0.234 **	
ELP × LCSE					−0.527 **
R2	0.035	0.142	0.152	0.188	0.278
F	5.963 **	18.327 **	17.017 **	25.646 **	36.519 **

Notes: * *p* < 0.05; ** *p* < 0.01; N = 672.

**Table 6:** Moderated mediation effect of OCC and LCSE.

Path	Mediator				Moderated Mediation	
	Moderator	Effect	SE	95% CI	Index	(CI)
ELP → Leader jealousy → Leader ostracism	Low OCC (M − 1SD)	0.096	0.037	[0.023, 0.166]	0.073	[0.006, 0.160]
	High OCC (M + 1SD)	0.188	0.042	[0.083, 0.203]		
	Difference group	0.092	0.049	[0.007, 0.201]		
	Low LCSE (M − 1SD)	0.173	0.030	[0.118, 0.232]	−0.177	[−0.242, −0.121]
	High LCSE (M + 1SD)	−0.004	0.025	[−0.055, 0.044]		
	Difference group	−0.177	0.031	[−0.241, −0.121]		

## Data Availability

The data presented in this study are available upon request from the corresponding author.
